# ADHD and overweight in boys: cross-sectional study with birth weight as a controlled factor

**DOI:** 10.1007/s00787-014-0531-1

**Published:** 2014-03-16

**Authors:** Tomasz Hanć, Agnieszka Słopień, Tomasz Wolańczyk, Monika Dmitrzak-Węglarz, Anita Szwed, Zbigniew Czapla, Magdalena Durda, Joanna Ratajczak, Joachim Cieślik

**Affiliations:** 1 Department of Human Biological Development, Faculty of Biology, Institute of Anthropology, Adam Mickiewicz University, Umultowska 89, 61-614 Poznan, Poland; 2 Department of Child and Adolescent Psychiatry, Poznan University of Medical Sciences, Poznan, Poland; 3 Department of Child Psychiatry, Medical University of Warsaw, Warsaw, Poland; 4 Laboratory of Psychiatric Genetics, Department of Psychiatry, Poznan University of Medical Sciences, Poznan, Poland

**Keywords:** ADHD, BMI, Birth body weight, Body size, Height, Overweight, Obesity, Weight

## Abstract

Population studies indicate a strong relationship between birth weight (BW) and body size in later life. However, BW as a variable was never accounted for in studies on the relationship between attention-deficit/hyperactivity disorder (ADHD) and overweight. This study aims to assess the relationship between ADHD and overweight with control of birth weight and other confounding factors. Prevalence of overweight was compared in clinical sample of 219 boys with ADHD and 396 boys without ADHD, aged 6–18 years. The following factors were controlled: BW, parents income and education level, place of residence, ADHD type, selected comorbid disorders and stimulant treatment. Overweight and obesity were diagnosed according to the criteria proposed by the International Obesity Task Force. Logistic regression analysis was used to estimate the association between ADHD and the prevalence of overweight and obesity. Boys with ADHD differed significantly from the control group in distribution of low BW (8.2 vs. 3.0 %, *χ* ^2^ = 8.23, *p* = 0.02). Low BW was associated with a lower prevalence of overweight than normal and high BW (0 vs. 12.14 %, *χ* ^2^ = 4.12, *p* = 0.04). Overweight was observed significantly more often in boys with ADHD (17.3 vs. 8.3 %, *χ* ^2^ = 11.23, *p* < 0.001) even after adjustment for BW and other variables (OR = 2.44, 95 % CI 1.38–4.29, *p* = 0.002) and after controlling for ADHD type, stimulant treatment and selected comorbid disorders. Independently to applied analysis, obesity was not associated with ADHD. Lower birth weight is over twice more often observed in boys with ADHD than in control group. Although this phenomenon may reduce the rate of overweight in the studied group, ADHD remains strongly associated with increased prevalence of overweight.

## Introduction

Increasing attention has been devoted to comorbidity between attention-deficit/hyperactivity disorder (ADHD) and overweight/obesity in the last few years. Studies suggest that ADHD may be a risk factor of excess body weight [1–3]. Researchers observe higher body weight or body mass index (BMI) of children and adolescents with ADHD in comparison with growth charts or control group of individuals without ADHD [4–9]. Additionally, a higher percentage of overweight and/or obesity was noted in children and adolescents with ADHD [5, 10–13]. The association between ADHD and overweight/obesity in children or adolescents has been demonstrated both in clinical [4–6, 8, 11, 13–17] and epidemiological studies [7, 10, 12, 18–20]. A similar relationship between ADHD and obesity was found in adults in epidemiological studies [21, 22]. ADHD is also diagnosed more often in clinically obese children [23], adolescents [24] and adults [25, 26]. Albayrak et al. [30] found a common genetic link between ADHD and obesity. However, some studies showed no relationship between body weight and ADHD [27–29].

Variables related to growth and body weight have to be controlled during a research on relationship between ADHD and obesity. These known factors include: (1) treatment with stimulants (that may cause a decrease in body weight and slow down growth) [16, 31–36], (2) ADHD comorbid conditions, e.g., depression (often associated with increased body weight, BMI, overweight and obesity) [14, 37], (3) place of residence (living in large urban agglomerations or rural areas may influence the risk of overweight) [38–41] and (4) socioeconomic status (both high and low status increases the risk of overweight and obesity) [42–47]. ADHD is also associated with low birth weight, a condition so prevalent in this group (compared to the whole population) that it serves as one of etiological factors for the disorder [48–52]. Until now, the relationship between the birth weight and body weight in children and adolescents with ADHD has never been evaluated, even though birth weight is related to body weight, BMI and level of fatness in later years of life [33–42].

High birth weight increases the risk of obesity and excess fatness, according to numerous studies [50, 53–57]. Low birth weight effect is not that evident. Some studies demonstrated that the low birth weight is a predictor of lower values of body weight, BMI and underweight in childhood and adolescence [58–62]. It is probably caused by parents’ genes—children’s body size highly correlates with body size of parents [63]. Other studies gave reverse results: small size at birth was associated with measures of later central obesity [64–68] as higher subcutaneous fat mass [69] and body fat percentage [70, 71], high ratio of subscapular to triceps skinfolds [72, 73], higher waist circumference [74], higher waist to hip ratio [75, 76], metabolic syndrome and insulin resistance [77–82] later in life. The association of low birth weight and subsequent obesity is explained by the programming or the fetal insulin hypothesis. The programming is understood as developmental adaptation of metabolism and physiology as a consequence of alterations in fetal nutrition [83]. Fetal programming contributes to the fact that low birth weight infants are more likely to have rapid growth as they catch up to normal; for some this fast growth becomes excessive and causes obesity [84]. The programming hypothesis is also supported by the fact, that people exposed to famine in late or middle period of gestation had, e.g., reduced glucose tolerance [85], atherogenic lipid profile [86] and higher BMI [87]. The fetal insulin hypothesis is based on the studies, which proves that impairments in fetal insulin secretion or sensitivity caused by genetic factors, such as, e.g., glutamate decarboxylase 2 gene overexpression, are associated with both: low birth weight and early onset obesity [83].

Taking into account the above considerations, one can hypothesize that the higher incidence of obesity in children, adolescents and adults with ADHD is associated with low birth weight, which is more frequently observed in this group. This would suggest that the relationship of ADHD to obesity can be explained, at least in part, by fetal programming hypothesis or the fetal insulin hypothesis.

The aim of our study was to assess the relationship between ADHD and prevalence of overweight and obesity in boys with control of their birth weight, subtype of ADHD, comorbid associated disorders, medication status and indices of socioeconomic status.

## Methods

### Ethical and financial statements

The study has been approved by the Ethics Committee of the Poznan University of Medical Sciences (Decision Number 855/05), and have therefore been performed in accordance with the ethical standards laid down in the 1964 Declaration of Helsinki and its later amendments. Study was also consistent with national law. The research received financial support from the Polish Ministry of Science and Higher Education (Grant Number NN303 0175 33). The subjects and their legal guardians were fully informed about research procedures and gave a written consent to participate in the study.

### Participants with ADHD

The research was carried out in Poland in the years 2005–2008. The examined group of children and adolescents with ADHD was recruited among patients of psychiatric hospitals and of university outpatient clinics. The study enrolled only boys because ADHD is diagnosed much less frequently in girls than in boys, thus the chances of gathering a representative clinical group of girls were small. The range of age of studied boys (6–18 years) was defined by the minimum age of patients diagnosed with ADHD in Poland and age they reach adulthood in accordance with Polish law. This wide age range gave the opportunity to assess the overweight and obesity rates in both populations—children and adolescents. Only boys without significant cognitive deficits (IQ ≥ 85) were enrolled to the study, for which the consent to participate in the study was obtained.

Participants had to have met the criteria for Diagnostic and Statistical Manual of Mental Disorders, 4th Edition, Text Revision (DSM-IV-TR) [88] for one of the three ADHD types: predominantly inattentive (ADD) or impulsive/hyperactive and combined type. Diagnosis was made by a team comprising of a psychiatrist, psychologist on the basis of the results of an interview with a parent, using the Diagnostic Structured Interview for ADHD and Hyperkinetic Disorder According to ICD-10 and DSM-IV-TR [89], Conners’ Parent Rating Scale and the observation of the patient behavior. When the results of all methods were consistent and indicated that the child meets the diagnostic criteria for ADHD, the diagnosis was considered confirmed. Only children with confirmed diagnosis were included in the studied group.

Exclusion criteria included: psychiatric disorders comorbid with ADHD, such as depression, anxiety disorder, bipolar affective disorder, anorexia or bulimia; endocrine disorders, such as diabetes, thyroid disease; which may, independently of ADHD, affect the body weight of the patients.

In this study we wanted to evaluate the association of birth weight with subsequent body weight of studied boys. Body weight at birth is strongly associated with the term of birth. Infants born prematurely have low birth weight and those postterm-born high birth weight. The term of birth, regardless of birth weight, is a factor associated with subsequent developmental disorders. Therefore, to examine the association of birth weight with subsequent obesity in boys with ADHD, undisturbed by the birth term, we decided to exclude boys preterm born (before 37 weeks of pregnancy) and postterm born (born after 42 weeks of pregnancy) from the study.

Birth weight and detailed information about delivery were obtained from medical registries. Then the subjects were categorized, according to guidelines of Centres for Disease Control [90], into three categories: with low birth weight (all the children with birth weight <2,500 g, without division into extremely low birth weight, very low birth weight and low birth weight), with normal birth weight (2,500–4,000 g) and high birth weight (>4,000 g).

The data about subjects’ socioeconomic status (SES) and health were collected using a questionnaire for parents, specially developed for the study. SES was assessed on the basis of two indices: parents’ income and education level. Parents were asked to assess their income level. Based on data from the report of Central Statistical Office, which includes average salaries in Poland in 2005–2008 [91], we considered the total income of parents in the range of 4,000–5,000 PLN netto as the average income. Higher income than 5,000 PLN was classified as above average and lower than 4,000 PLN as below average. Parents were divided into three categories due to their level of education: both without higher education, one with higher education, both with higher education. Although income and education level are the indicators of SES in the study, we chose not to calculate the total index. We have taken into account the possibility that these variables may be related to body weight of children also in other ways. A higher level of income may be associated with better access to food, access to food of higher quality, but also to a better health care. Higher level of parental education, regardless its relationship to SES, may also be associated with a greater health awareness of parents and more health-oriented model of upbringing.

Subjects were divided according to place of residence into two categories: those living in a large cities with more than 500,000 inhabitants, and those from smaller towns and rural areas. We have chosen this criteria because in Poland university centers and psychiatric clinics are found mainly in cities exceeding 500,000 inhabitants. In smaller towns and rural areas, the opportunity to benefit from the aid of children’s psychiatrist, and hence, obtaining a diagnosis of ADHD is limited. Furthermore, population density of the place of residence is an important factor associated with the development and rate of body fat. We were aware that boys inhabiting large cities could be more prone to overweight, because of their more sedentary lifestyle. This could falsify our results by strengthening the relationship between ADHD and overweight.

Data about ADHD treatment came from medical registries maintained in outpatient clinics. However, data about treatment history, used medications, their doses and time of administration were not available for the whole sample. Therefore, the group of *currently medicated* patients included subjects who received stimulants at the time of the study, while the group of *not currently medicated* subjects included all the children who did not receive stimulants at the time.

Measurements of body height were carried out by trained medical staff with the use of an anthropometer (Gneupel Praezision Mechanik, Swiss) with measurement accuracy of ±1 mm. Measurements were made according to the standard technique [92–94]. Body weight was measured with a medical scale (Radwag PUE c/31) with measurement accuracy of ±100 g. The examined children were wearing only underwear. In order to minimize measurement error, two measurements of each individual were carried out during the same visit, and arithmetic means were considered as the actual values of height and body weight. The measurements were performed between 8 am and 4 pm.

BMI was calculated on the basis of height and body weight. Body height and BMI were later adjusted for sex and weight on the basis of World Health Organization (WHO) growth charts [95] and presented as *z* scores.

International Obesity Task Force (IOTF) recommendation [96] was used for the identification of individuals with overweight, obesity and underweight. IOTF cut-off points for subsequent age categories, indicated on the basis of BMI data from six nationally representative surveys, corresponded approximately to 90th percentile for overweight, 98th percentile for obesity and 3rd percentile for underweight (grade 2 of thinness) in those datasets [96, 97].

### Control group

For the control group, we have used the data collected for the purpose of our second study on the prevalence of overweight in children and adolescents in Poland. Both studies were conducted simultaneously. First, we have gathered the data from boys and girls aged 6–18 years; the recruitment was conducted in primary and secondary schools. Children were included in the study only after submission of the written consent of their parents or legal guardians. The research included anthropometric assessment of height and body weight (the procedure of measurement and data processing were the same as in the clinical trial), questionnaires and medical registries for collection of demographic, socioeconomic and perinatal data, as well as data concerning health of the subjects. Assessment of mental health was based on parents’ responses to the following questions: (1) ‘Has a doctor or health professional ever told you that your child has attention deficit disorder, attention-deficit/hyperactive disorder that is Attention Deficit Disorder (ADD) or ADHD?’ (2) ‘Has a doctor or health professional ever told you that your child has psychiatric disorders?’ (3) ‘Has a doctor or health professional ever told you that your child has oppositional defiant disorder, conduct disorder, emotional problems, such as depression or anxiety disorder?’ Similar questions were used in earlier studies for identification of individuals with and without ADHD [12, 98, 99].

Boys aged 6–18 years, term-born, inhabiting a region of the same urbanization level as the examined boys with ADHD, without endocrine diseases, whose parents had answered ‘no’ to the above questions, and for whom complete data were collected, were assigned as the control group. Differences between boys with ADHD and those of normal development, selected based on above-mentioned criteria, were analyzed in the study.

### Data analyses

Two-tailed *t* test was used for comparisons of boys currently medicated and not medicated as well as for comparisons of children with ADHD and the control group in terms of *z* scores for height and BMI, birth weight and parents’ education. *χ* ^2^ test was used for assessment of differences in prevalence of underweight, overweight and obesity as well as of categories of birth weight, age and education of parents, place of residence and parents’ income level. Then it was determined if variables that differed between both examined groups were associated with higher prevalence of overweight. *χ* ^2^ test was also used for this purpose.

Stepwise logistic regression with quasi-Newton estimation method was used to assess the probability of overweight/obesity and to control for other factors. The analysis was carried out for the whole sample and then separately: for patients with combined type of ADHD; for patients with comorbid oppositional defiant disorder and conduct disorder; as well as for patients not treated pharmacologically at the time. In all cases, two types of models were used: unadjusted and adjusted for all the variables, which statistically significantly differed boys with ADHD and control group.

We also evaluated an association between ADHD as a single factor and prevalence of overweight in the subgroup selected with inclusion criteria: normal or high birth weight, average income level, family with one parent with higher education, place of residence in large cities (above 500,000 citizens), not taking stimulants at the time of the research and without associated oppositional defiant disorder and conduct disorder.

In all the analyses, results were considered statistically significant at significance level *p* < 0.05. Statistical analyses were conducted with Statistica 10 software. Height and BMI were transformed into *z* scores with WHO AnthroPlus software.

## Results

### Characteristics of the sample

Consent to the research was obtained for 296 boys with diagnosed ADHD. The following boys were excluded from the group: 6 boys with depression, 11 boys with anxiety disorder, 24 preterm-born boys, 28 postterm-born ones, and 8 boys for whom incomplete data were collected. Among the initial number of 784 boys applying for the control group, the following ones were excluded: 82 boys—due to the risk of mental disorders, 167 preterm-born boys and 5 postterm-born boys, and 7 individuals with hormonal disturbances. One hundred twenty-seven individuals were excluded due to incomplete data.

In the end, the analysis included 219 boys with ADHD and 396 boys in control group. Among the boys with ADHD, combined type of the disorder was predominant (*n* = 161, 73.5 %). Most of the group (*n* = 167, 76.3 %) had at least one additional disorder, and the most prevalent ones were learning disorders (*n* = 59, 26.9 %) and oppositional defiant disorder (*n* = 55, 25.1 %). Less than half of the sample (*n* = 79, 36.1 %) was treated with stimulants at the time of the research. Boys not currently medicated and currently medicated did not differ in terms of BMI, prevalence of underweight, overweight and obesity as well as other controlled variables (Tables 1, 2).

**Table 1:** Characterization of boys with ADHD

Characteristics	Categories	*n*	%
Type of ADHD			
	ADD	37	16.9
	Hyperactive/impulsive	21	9.6
	Combined	161	73.5
Comorbid disorders			
	At least one additional diagnosed disorder	167	76.3
	Learning disorders	59	26.9
	Speech disorders	31	14.1
	Tic disorder	7	3.2
	Oppositional defiant disorder	55	25.1
	Conduct disorder	15	6.8
Treatment			
	Not currently medicated	140	63.9
	Currently medicated	79	36.1

*ADD* attention deficit disorder, *n* number, % percentage

**Table 2:** Comparison between groups of boys with ADHD distinguished based on medication status

Characteristics	Not currently medicated (*n* = 140) Mean ± 1SD or *n* (%)	Currently medicated (*n* = 79) Mean ± 1SD or *n* (%)	Differences between groups (2-tailed *t* test or *χ* ^2^ and *p*)
Age	11.2 ± 2.8	11.1 ± 2.5	*t* = 0.09, *p* = 0.92
Height (sex and age adjusted)	0.6 ± 1.2	0.5 ± 1.2	*t* = −0.50, *p* = 0.62
BMI (sex and age adjusted)	0.4 ± 1.4	0.4 ± 1.4	*t* = −0.25, *p* = 0.80
Underweight rates	3 (2.1)	2 (2.5)	*χ* ^2^ = 0.03, *p* = 0.85
Overweight rates	25 (17.9)	13 (16.5)	*χ* ^2^ = 0.07, *p* = 0.79
Obesity rates	6 (4.3)	5 (6.3)	*χ* ^2^ = 0.44, *p* = 0.51
Overweight or obesity	31 (22.4)	18 (22.8)	*χ* ^2^ = 0.01, *p* = 0.91
Body weight at birth (g)	3,294 ± 663.15	3,409 ± 530.50	*t* = −1.33, *p* = 0.19
Low birth weight	18 (8.2)	12 (3.0)	*χ* ^2^ = 2.20, *p* = 0.33
Normal birth weight	115 (82.1)	66 (83.5)	
High birth weight	11 (7.9)	9 (11.39)	
Mother’s age at birth (years)			
<25	51 (36.4)	35 (44.3)	*χ* ^2^ = 1.98, *p* = 0.37
25–35	74 (52.9)	39 (49.4)	
>25	15 (10.7)	5 (6.3)	
Level of parents’ education			
Both with education below higher	4 (2.9)	4 (5.1)	*χ* ^2^ = 0.73, *p* = 0.69
One with higher education	85 (60.71)	46 (58.23)	
Both with higher education	51 (36.43)	29 (36.71)	
Place of residence			
City with over 500,000 citizens	119 (85.0)	72 (91.1)	*χ* ^2^ = 1.71, *p* = 0.19
Parents’ income level			
Below average	13 (9.3)	4 (5.1)	*χ* ^2^ = 2.80, *p* = 0.25
Average	58 (41.4)	41 (51.9)	
Over average	69 (49.3)	34 (43.0)	

Boys with ADHD and control group were at the same age: mean age for boys with ADHD was 11.2 years ± 2.7, and for boys from control group 10.8 years ± 2.8 (*t* = −1.64, *p* = 0.10). In the both groups, mother’s age at the time of childbirth was most often in the range of 25–35 years (51.6 vs. 57.1 %, *χ* ^2^ = 1.77, *p* = 0.41). Compared groups differed in terms of the other controlled variables. In the both groups, families with *one parent with higher education* were the most numerous, though a higher percentage of *both parents with higher education* class was observed in boys’ with ADHD families in comparison with control group (36.5 vs. 18.2 %, *χ* ^2^ = 27.94, *p* < 0.001). More boys with ADHD lived in large cities with above 500,000 citizens (87.2 vs. 65.3 %, *χ* ^2^ = 61.02, *p* < 0.0001). More parents of boys with ADHD were declared above-average income, compared to the control group (47.0 vs. 11.1 %, *χ* ^2^ = 103.03, *p* < 0.001).

No statistically significant differences between means of birth weight were found (3,336 vs. 3,409 g, *t* = −1.61, *p* = 0.12). However, categorization into low, normal and high birth weight demonstrated that boys with ADHD over twice more often had low birth weight (8.2 vs. 3.0 %, *χ* ^2^ = 8.23, *p* = 0.02).

*t* test showed no statistically significant differences in sex and age-adjusted height and BMI (for height *t* = 1.54, *p* = 0.12 and for BMI *t* = 0.39, *p* = 0.69). After distinguishing body weight categories with the method recommended by IOTF, significantly higher percentage of overweight/obesity (as one category) was noted in boys with ADHD (ADHD: 22.4 vs. control group: 13.9 %, *χ* ^2^ = 7.23, *p* = 0.007) when compared to the control group. More detailed analysis revealed that the prevalence of overweight was over twice higher in boys with ADHD (ADHD 17.3 % vs. control group 8.3 %, *χ* ^2^ = 11.23, *p* < 0.001), while the prevalence of obesity in the both groups was similar (ADHD 5.0 %, control group 5.6 %, *χ* ^2^ = 0.08, *p* = 0.78). The compared groups did not differ in terms of prevalence of underweight (ADHD 2.3 %, control group 1 %, *χ* ^2^ = 1.58, *p* = 0.21).

We investigated if birth weight was associated with *z* scores for BMI. Analyses were conducted for the whole sample, without division into the group of boys with ADHD and control group. *z* scores for BMI of the subjects with low birth weight was −0.01. For boys with normal birth weight, *z* score for BMI was 0.36 and for those with high birth weight was 0.40. Differences between mean BMI were examined for two groups: boys with low birth weight and boys with normal or high birth weight. The differences were not statistically significant (*t* = −1.60, *p* = 0.11). Overweight was not observed in boys with low birth weight, while the prevalence of overweight in boys with normal and high birth weight was, respectively, 12.1 and 12.5 %. The difference in the prevalence of overweight between the two groups of boys: boys with low birth weight and boys with normal or high birth weight, was statistically significant (0 vs. 12.1 %, *χ* ^2^ = 4.12, *p* = 0.04). Obesity was diagnosed in 6.7 % of boys with low birth weight, 5.3 % of boys with normal birth weight and 5.4 % of boys with high birth weight. The difference in obesity rate between boys with low birth weight and the rest of the sample was not statistically significant (6.7 vs. 5.3 %, *χ* ^2^ = 0.10, *p* = 0.75).

There were no statistically significant differences (*t* = −0.25, *p* = 0.80) in *z* scores for BMI between currently medicated (*z* = 0.41) and untreated boys with ADHD (*z* = 0.36). Underweight occurred in 2.53 % of currently medicated boys and in 2.14 % of boys untreated (*χ* ^2^ = 0.03, *p* = 0.85). Overweight was found in 16.46 % of boys currently medicated and in 17.86 % of untreated boys (*χ* ^2^ = 0.07, *p* = 0.79). Obesity occurred in 6.33 % of boys currently medicated and in 4.29 % of untreated (*χ* ^2^ = 0.44, *p* = 0.51). Differences between groups were not statistically significant.

*χ* ^2^ test did not show significant differences in the prevalence of overweight between categories of the other variables that differed the compared groups of boys with ADHD and control group, i.e., parents’ income and education level, place of residence (Table 3).

**Table 3:** Comparison of boys with and without ADHD

Characteristics	Boys with ADHD (*n* = 219) Mean ± 1SD or *n* (%)	Boys without ADHD (*n* = 396) Mean ± 1SD or *n *(%)	Differences between groups of boys with and without ADHD (2-tailed *t* test or *χ* ^2^ and *p*)	Differences in rates of overweight between categories of independent variables (*χ* ^2^ and *p*)
Age	11.2 ± 2.7	10.8 ± 2.8	*t* = -1.64, *p* = 0.10	
Height (sex and age adjusted)	0.6 ± 1.2	0.4 ± 1.2	*t* = 1.54, *p* = 0.12	
BMI (sex and age adjusted)	0.4 ± 1.4	0.3 ± 1.2	*t* = 0.39, *p* = 0.69	
Underweight rates	5 (2.3)	4 (1.0)	*χ* ^2^ = 1.58, *p* = 0.21	
Overweight rates	38 (17.3)	33 (8.3)	***χ*** ^**2**^ **=** **11**.**23**, ***p*** **<** **0**.**001**	
Obesity rates	11 (5.0)	22 (5.6)	*χ* ^2^ = 0.08, *p* = 0.78	
Overweight or obesity	49 (22.4)	55 (13.9)	***χ*** ^**2**^ **=** **7**.**23**, ***p*** **=** **0**.**007**	
Body weight at birth (g)	3,336 ± 619.83	3,409 ± 486.97	*t* = 1.61, *p* = 0.12	
Low birth weight	18 (8.2)	12 (3.0)	***χ*** ^**2**^ **=** **8**.**23**, ***p*** **=** **0**.**02**	***χ*** ^**2**^ **=** **4**.**12**, ***p*** **=** **0**.**04**
Normal birth weight	181 (82.7)	348 (87.9)		
High birth weight	20 (9.1)	36 (9.1)		
Mother’s age at birth (years)				
<25	86 (39.3)	140 (35.3)	*χ* ^2^ = 1.77, *p* = 0.41	
25–35	113 (51.6)	226 (57.1)		
>25	20 (9.1)	30 (7.6)		
Level of parents’ education				
Both with education below higher	8 (3.7)	34 (8.6)	***χ*** ^**2**^ **=** **27**.**94**, ***p*** **=** **0**.**001**	*χ* ^2^ = 0.94, *p* = 0.63
One with higher education	131 (59.8)	290 (73.2)		
Both with higher education	80 (36.5)	72 (18.2)		
Place of residence				
City with over 500,000 citizens	191 (87.2)	223 (56.3)	***χ*** ^**2**^ **=** **61**.**02**, ***p*** **<** **0**.**001**	*χ* ^2^ = 3.76, *p* = 0.05
Parents’ income level				
Below average	17 (7.8)	81 (20.5)	***χ*** ^**2**^ **=** **103**.**03**, ***p*** **<** **0**.**001**	*χ* ^2^ = 2.20, *p* = 0.33
Average	99 (45.2)	271 (68.4)		
Over average	103 (47.0)	44 (11.1)		

*n* Number, % percentage, *SD* standard deviation, *t* value of Student’s *t* test, *χ* ^*2*^ value of *χ* ^2^ test, *p* significance level, bold significant differences

### Assessment of the relationship between ADHD and overweight/obesity

Logistic regression was used to assess the association between ADHD and overweight. ADHD was statistically significantly related to higher rate of overweight, when ADHD was treated as a single factor and in models taking into consideration the influence of birth weight, place of residence, parents’ education and income level [unadjusted model OR (odds ratio) = 2.31, 95 % CI 1.40–3.81, *p* = 0.001; adjusted model: OR = 2.44, 95 % CI 1.38–4.29, *p* = 0.002]. The influence of birth weight (OR = 1.68, 95 % CI 0.88–3.17, *p* = 1.68) and the other variables included in the model was not statistically significant.

Separate models were analyzed for: (1) boys with only combined type of the disorder (*n* = 161), (2) boys with ADHD with exclusion of individuals with associated oppositional defiant disorder and conduct disorder (*n* = 149), and (3) boys not treated with stimulants at the time (*n* = 140), to control the influence of these characteristics in the group of boys with ADHD. Control group was the reference in all the models. Values of OR for ADHD factor differed between the models (unadjusted models OR 2.11–2.62; adjusted models OR 2.17–2.71), however, ADHD still remained the only variable that was statistically significantly related to overweight (unadjusted models *p* <0.001–0.009; adjusted models *p* <0.0001–0.02) (Table 4).

**Table 4:** Results of logistic regression analysis: association between ADHD and overweight in unadjusted and adjusted models

	ADHD, the whole group		ADHD, combined type		ADHD with exclusion of children with oppositional defiant disorder and conduct disorder		ADHD, currently not medicated	
	OR (95 %CI)	*p*	OR (95 %CI)	*p*	OR (95 %CI)	*p*	OR (95 %CI)	*p*
ADHD, unadjusted	**2**.**31** (**1**.**40–3**.**81**)	**0**.**001**	**2**.**62** (**1**.**54–4**.**46**)	**<0**.**001**	**2**.**11** (**1**.**20–3**.**72**)	**0**.**009**	**2**.**42** (**1**.**46–4**.**01**)	**<0**.**001**
ADHD, adjusted for:	**2**.**44** (**1**.**38–4**.**29**)	**0**.**002**	**2**.**71** (**1**.**49–4**.**96**)	**0**.**001**	**2**.**17** (**1**.**15–4**.**08**)	**0**.**02**	**2**.**57** (**1**.**46–4**.**56**)	**<0**.**001**
Birth weight	1.68 (0.88–3.17)	1.68	1.99 (0.98–4.02)	0.05	1.64 (0.80–3.33)	0.17	1.65 (0.86–3.17)	0.13
Parents’ education	1.12 (0.67–1.86)	0.67	1.18 (0.67–2.07)	0.57	1.10 (0.62–1.94)	0.74	1.18 (0.70–1.97)	0.53
Place of residence	1.38 (0.73–2.60)	0.31	1.20 (0.63–2.30)	0.57	1.52 (0.78–2.99)	0.22	1.36 (0.72–2.56)	0.34
Income level	0.73 (0.47–1.14)	0.16	0.84 (0.51–1.39)	0.50	0.70 (0.42–1.17)	0.17	0.70 (0.45–1.10)	0.12

*n* number, *OR* odds ratio, *95 CI* 95 % confidence interval, *p* significance level, *bold* significant effects

The same models were used to test the relationship between ADHD and obesity. All the analyses showed a lack of statistically significant correlation between the incidence of ADHD and obesity, regardless of what variables were controlled in the models (Table 5).

**Table 5:** Results of logistic regression analysis: association between ADHD and obesity in unadjusted and adjusted models

	ADHD, the whole group		ADHD, combined type		ADHD with exclusion of children with oppositional defiant disorder and conduct disorder		ADHD, currently not medicated	
	OR (95 % CI)	*p*	OR (95 % CI)	*p*	OR (95 % CI)	*p*	OR (95 % CI)	*p*
ADHD, unadjusted	0.90 (0.43–1.89)	0.78	0.66 (0.26–1.66)	0.37	0.84 (0.35–2.01)	0.69	0.76 (0.30–1.92)	0.56
ADHD, adjusted for:	0.79 (0.34–1.83)	0.58	0.63 (0.23–1.72)	0.37	0.84 (0.32–2.18)	0.71	0.72 (0.26–1.97)	0.52
Birth weight	0,84 (0.12–5.58)	0.86	0.46 (0.05–4.28)	0.49	0.43 (0.05–3.63)	0.44	0.91 (0.11–7.69)	0.93
Parents’ education	0.48 (0.11–2.22)	0.35	0.77 (0.14–4.25)	0.77	0.78 (0.15–3.98)	0.76	0.87 (0.17–4.54)	0.87
Place of residence	1.22 (0.54–2.74)	0.63	1.06 (0.46–2.44)	0.89	1.10 (0.48–2.53)	0.82	1.09 (0.48–2.51)	0.83
Income level	1.79 (0.45–7.15)	0.86	1.08 (0.23–5.09)	0.92	0.97 (0.21–4.51)	0.97	1.21 (0.28–5.24)	0.80

*n* number, *OR* odds ratio, *95 CI* 95 % confidence interval, *p* significance level

Additionally, the association of ADHD and overweight was assessed in the subgroup created on the basis of normal or high birth weight, average income level, one parent with higher education, living in a large city, not receiving stimulants at the time of the research and without associated oppositional defiant disorder and conduct disorder. The analysis included 24 patients with ADHD and 139 boys of control group. Overweight was observed in 7 (29.17 %) boys with ADHD and in 11 (7.91 %) subjects without ADHD. Logistic regression analysis revealed that ADHD remained a factor that was statistically significantly associated with higher rate of overweight, and OR in thus selected group increased to 4.79 (95 % CI 1.63–14.14, *p* = 0.004).

## Discussion

This research assessed the relationship between ADHD and overweight/obesity in children and adolescents with control of birth weight and also socioeconomic status, treatment status, ADHD type, selected comorbid disorders and. When comparing our results with other studies on the relationship between ADHD and overweight/obesity in children and adolescents, we had to be cautious due to differences in the methodology and terminology. Some researchers use the criteria for overweight and obesity proposed by the US Centres for Disease Control and Prevention (CDC), which define the cut-off point for overweight as the 85th percentile and 95th percentile for obesity [e.g., 10, 12, 13, 100, 101], other authors use percentile 90th and 97th [2, 20] or 85th and 97th [102]. Even with identical criteria adopted according to CDC, some authors define the 85th percentile as a cut-off point for at risk for overweight and the 95th percentile for overweight [12, 100]. Thus, the terms overweight and obesity are often used synonymously. In our study, we have adopted the guidelines of International Obesity Task Force, in which the cut-off points for subsequent age categories correspond approximately to 90th percentile for overweight and 98th percentile for obesity in the population of the world [96]. When comparing our results with results of earlier studies, we will use the terms: overweight for BMI > 85th percentile or percentile >90th, and obesity for BMI > 95th percentile or percentile >97th.

Our study indicates that ADHD is significantly associated with higher prevalence of overweight, but not obesity in boys. ADHD, depending on assumed model (with control of confounding variables) was associated with increased prevalence of overweight in boys from 2.17 to 2.71 times. This agrees with the results of Erhart et al. [20], which showed significant differences in the incidence of overweight (≥90th percentile) between children with and without ADHD (19.8 vs. 10 %) and much lower in frequency of obesity (≥97th percentile 8.8 and 7.6 %, respectively). Similarly, Rojo et al. [103] showed no significant association of ADHD with obesity, while Holtkamp et al. [5], using the same criteria, proved the existence of revealed a relationship between ADHD and both overweight and obesity. Divergent results were obtained also for cut-off points of 85 and 95th percentile. Using these criteria, some studies have shown the relationship of ADHD with overweight and obesity [12] while others have not [100]. Lam and Yang [10] demonstrated a correlation of ADHD with obesity, but not with overweight. Kim et al. [101] evaluated the relationship between obesity and ADHD and showed statistically significant results for not-medicated children. The study of Egmond-Fröhlich et al. [104], which adopted the same IOTF criteria that we have used, revealed connection between high severity symptoms of hyperactivity/inattention with a higher incidence of overweight/obesity as a combined category. As shown above, differences between the results of studies exist regardless of adopted classification of overweight and obesity. This may be a result of other differences in the methodology, such as inclusion and exclusion criteria, age and sex of respondents, the status of the treatment of the children, controlled coexisting disorders and accompanying socioeconomic and cultural variables.

Several factors, which could have influenced our results, were controlled in the study. For us, the most interesting variable to examine was the birth weight, so far never taken into consideration in studies on the relationship between ADHD and overweight/obesity. We observed that low birth weight was over twice more frequent in boys with ADHD than in the control group, which agrees with available data [48, 49, 51, 52]. In the examined groups, no cases of overweight were noted in children with low birth weight, while in children with normal and high birth weight the prevalence of overweight was 12.14 %. It agrees with the studies demonstrating a relationship between low birth weight and small body weight in childhood and adolescence [58–62], and contradicts the results suggesting that low birth weight may be a predictor of a high level of fatness and overweight in later life [80–85]. However, due to the fact that 6.7 % of the group of boys with low birth weight was obese, it is not possible to determine the final one-way relationship between birth weight and later body weight. We hypothesize that fetal programming effect or the fetal insulin hypothesis (associated with low birth weight) do not explain the strong relationship of ADHD and overweight. On the contrary, our study may suggest that higher frequency of children with low birth weight among patients with ADHD weakens the association of ADHD and excessive body weight.

ADHD remained the only factor related to overweight after adjusting for several other variables, which are usually associated with the level of BMI, i.e., place of residence [14, 37–41] and indices of socioeconomic status, such as income or parents’ education [42–47]. Some studies on the association between ADHD and overweight demonstrated that low level of SES was associated with a higher risk of overweight in children with ADHD [12, 100]. Although we expected similar results, in our case indices of SES were not related to the prevalence of overweight.

Earlier studies found that depression and anxiety disorder may increase prevalence of overweight/obesity in individuals with ADHD [3, 12, 14, 37]. Therefore, we excluded patients with these disorders from our study. However, we kept the potential influence of oppositional defiant disorder and conduct disorder on the obtained results under control. Some earlier studies indicated a relationship between these disorders and height and body weight of children, adolescents and adults [105–108]. Nevertheless, in our research, ADHD was significantly associated with overweight also when the analysis included only boys without the above-mentioned disorders.

The relationship between ADHD and overweight remained significant when children with full-blown ADHD (combined type) or those not treated with stimulants at the time were selected for the analyses. Earlier papers demonstrated a decrease in body weight and inhibition of growth due to treatment [12, 16, 31–36, 104]. Therefore, one should expect that after exclusion of patients undergoing treatment, the demonstrated relationship between ADHD and overweight not only will remain significant, but will be strengthened. The results met our expectations. Odds ratio for overweight were slightly higher when the patients not treated were compared with the control group, than when we analyzed the whole group of boys with ADHD, regardless of their medication status. The incidence of obesity did not differ between boys from the groups that were distinguished due to medication status.

An additional analysis was conducted to examine how ADHD increases the risk of overweight in a group thoroughly selected in terms of controlled variables. Since low birth weight could have decreased the obtained indexes, only the data concerning boys with normal and high birth weight were used in the analysis. Additional inclusion criteria were average income level, average level of education of parents, inhabiting large urban agglomerations, no comorbid oppositional defiant disorder and conduct disorder, and not receiving stimulants at the time of the research. Such a selection enabled the assessment of ‘pure’ association of ADHD and the prevalence of overweight. We demonstrated that the diagnosis of ADHD is associated with nearly five-time higher risk of overweight than it is in case of healthy children. However, the result should be approached with caution due to the small number of individuals meeting the criteria for inclusion in the selected homogeneous group (boys with ADHD *n* = 24, reference group *n* = 139).

The presented research has its limitations. The study left out few variables, which may result in increased body weight of children with ADHD. We did not analyze sleep disorders, lifestyle, physical activity and diet. It was also not possible to analyze treatment history more thoroughly. We did not have complete data on the doses and time of administration of medications. To the group of patients not treated we qualified boys, who were not taking medication at the time of the study. The study does not consider how much time has elapsed since discontinuation of treatment of the boys, who were previously treated with stimulants. Similarly, a group of boys under treatment enrolled patients who received medication at the time of the study, but we did not have information when the treatment started. Adopted methodology for distinguishing medication status limits our study. This may explain why we did not observe differences in the level of body height and BMI between boys included in both the groups (treated and not treated), even though previous work showed the influence of stimulant treatment on the growth of children with ADHD [16, 31–36].

Some limitations refer to the criteria of inclusion. We did not use information gathered from the teachers to confirm diagnosis of ADHD in the sample. Teachers were requested to fill out the questionnaires on ADHD symptoms, but unfortunately most of them did not return the questionnaire. It was not possible also to give more precise psychiatric diagnosis of the control group. We believe that the questions, similar to those of earlier large population studies [12, 60, 61], enabled exclusion of individuals with symptoms of the disorders in question. We agree that this method, although accepted and used in population studies, is not equal to clinical diagnosis made for children with ADHD.

The assessment of socioeconomic status was made only on the basis of two separate indicators: assessment of the income and education level of parents. Hence, our methods did not allow a completely objective and accurate assessment of SES. In our opinion, however, they provide an important insight on the quality of condition of the children’s growth and the differences in this area between the studied boys with ADHD and a control group.

We did not evaluate the sexual maturation stage, while other studies indicated a significant relationship between increased prevalence of overweight and the onset of puberty [13]. Finally, the conclusions of the study are limited to boys.

We left out a few factors, which might explain the relations between ADHD and overweight in the present study. Apart from common genetic background of ADHD and obesity [2, 30], the literature names social exclusion of children, inadequate diet, emotional eating, sedentary lifestyle, hours spent watching TV/day, problems with behavior regulation and control of executive functions as potential intermediary factors [2, 12, 100–102, 109].

Presented research proved that ADHD is significantly related to increased frequency of overweight in boys irrespective of birth weight, as well as of income level and education of parents, place of residence, ADHD type, medication status and selected comorbid disorders. Low birth weight is twice more frequent in boys with ADHD than in control group, though fetal programing or the fetal insulin hypothesis fails to explain association between ADHD and overweight. Low birth weight may decrease the prevalence of overweight in the group, therefore this factor should be under control in future studies.
